# Editorial: Addressing community priorities in autism research

**DOI:** 10.3389/fpsyg.2022.1040446

**Published:** 2022-09-27

**Authors:** Amy Pearson, Andrew Surtees, Catherine J. Crompton, Craig Goodall, Dhanya Pillai, Felicity Sedgewick, Sheena K. Au-Yeung

**Affiliations:** ^1^Faculty of Health and Wellbeing Sciences, School of Psychology, University of Sunderland, Sunderland, United Kingdom; ^2^School of Psychology, University of Birmingham, Birmingham, United Kingdom; ^3^Birmingham Women's and Children's National Health Service (NHS) Foundation Trust, Birmingham, United Kingdom; ^4^The Division of Psychiatry, The Salvesen Mindroom Research Centre, Centre for Clinical Brain Sciences, The University of Edinburgh, Edinburgh, United Kingdom; ^5^St Mary's University College, Queen's University, Belfast, United Kingdom; ^6^Ara Damansara Medical Centre, Selangor, Malaysia; ^7^School of Education, University of Bristol, Bristol, United Kingdom; ^8^Clinical Psychology Unit, Department of Psychology, The University of Sheffield, Sheffield, United Kingdom

**Keywords:** mental health, epistemic (in)justice, autism, education, participatory design, double empathy theory

Autism is a form of neurodiversity, currently characterized by differences compared to the neurotypical population across multiple domains including sensory processing (Proff et al., 2021), social communication style (Crompton et al., 2021), attentional processing (Murray et al., 2005), and movement and motor processing (Miller et al., 2021). Historically, autism (and thus autistic people) has been studied through a medical lens (Chapman and Carel, 2022), owing primarily to the characterization of autism as a disorder of childhood development. These conceptualizations led to dehumanizing narratives about autistic people (Botha) and have impacted on who we consider to be knowledgeable about what it is like to be autistic (Kourti). In recent years, there has been a shift toward recognition of autism as a form of neurodivergence; a naturally occurring variation in the human population that may lead to a differential profile of strengths and challenges in comparison to the non-autistic population (Den Houting, 2019). This shift has been primarily driven by the autistic self-advocacy and neurodiversity movements (Kapp et al., 2013; Walker, 2021), which have campaigned for better understanding of autistic people.

The push for a better understanding has included a demand for research which better serves autistic people and their priorities (Poulsen et al., 2022). In 2013, a report from Pellicano et al. (2014) revealed that whilst the majority of funding in autism research was allocated toward genetic profiling and biomedical intervention, very little went toward what community stakeholders (including autistic people and their family members) saw as valuable research. There was a strong desire amongst the autistic and broader autism communities for an increase in research, and associated support outcomes, in areas such as physical and mental healthcare, education, and employment (see also James Lind Alliance, 2016). In what is now almost a decade since that report was released, we have seen a sharp increase in research that addresses these autistic community priorities.

One way that this has been achieved has been through participatory research, whereby community members and stakeholders engage in developing research in consultation and collaboration (Keating; den Houting et al.) with researchers. Involving autistic people in research about them can shape more ethical and impactful research, as outlined by Keating in his opinion article on how participatory autism research can benefit everyone. However, we still have a way to go. den Houting et al. found that research stakeholders feel that academics are still disconnected from the communities they serve, and have a tendency to tokenize the input of community members when developing research. These sentiments are also compounded by the dehumanizing narratives surrounding autistic people which can make engaging in research as both a community member and a researcher a painful experience as outlined by Botha. For Kourti, the solution requires more than a participatory approach. They argue for the importance of autistic-led theory and practice in autism research, drawing upon a critical realist framework (Bhaskar, 1987) to emphasize how embodied knowledge of what it is like to be autistic can produce more credible work. These articles provide us with a way forward for meaningful autism research: non-autistic researchers need to recognize the burden that autistic people face in engaging with autism research, and work to create a more hospitable (and credible) field for all.

One example of an autistic-led theory which has garnered much empirical support is the Double Empathy Problem (Milton, 2012). Milton proposes that it is not autistic “social deficits” that underlie communication breakdowns between autistic and non-autistic people, but significant differences in how autistic and non-autistic people experience and process the world around them, and a lack of reciprocal understanding between the two groups. Thus, social communication is not a difficulty experienced solely by an autistic person, but a “double problem” that is experienced within an interaction between an autistic and non-autistic person (Davis and Crompton, 2021). Non-autistic people experience similar difficulty in understanding autistic people as autistic people do in understanding non-autistic people (Chown, 2014; Edey et al., 2016; Sheppard et al., 2016; Crompton et al., 2020).

Several papers in this special issue are centered on the concept of Double Empathy, and innovative ways to embody its principles to improve communication between autistic and non-autistic people. Whilst most social interventions for autism are targeted at autistic people, Jones et al. piloted a brief autism acceptance training aimed at non-autistic people to enhance their understanding of autistic people. They then compared dyadic interactions between (i) non-autistic people who had completed the training and autistic people, and between (ii) non-autistic people who had not completed the training and autistic people. In the dyads where the non-autistic person had completed the training, both the non-autistic and the autistic person expressed greater interest in spending social time together in the future. This promising finding suggests that increasing non-autistic people's understanding of autism may minimize the social exclusion faced by autistic people. Chapple, Davis, Billington, Williams, et al. and Chapple, Davis, Billington, Myrick, et al. used a novel approach to examine the facilitation of empathy between autistic and non-autistic partner dyads. Participants read *Of Mice and Men* (Steinbeck, 1937), before completing reading diaries, a creative writing task, and discussing the book with their partner. In Chapple, Davis, Billington, Williams, et al. autistic participants showed enhanced socio-empathic interpretations of the novel compared to the non-autistic participants. In Chapple, Davis, Billington, Myrick, et al. non-autistic participants reported an enhanced understanding of what it means to be autistic, while the autistic group reported feeling valued by their non-autistic reading partners and overcame their worries about non-autistic stereotypes of autism. Working together to appreciate each other's differences and experiences facilitated mutual understanding between autistic and non-autistic people.

Two further papers consider how the double empathy problem may play out in education and social support. Brownlow et al. highlight the crucial role that effective communication between teachers and autistic students plays in supporting successful school participation. Rather than depending on assumptions and stereotypes of autism, pupils wanted teachers to ask them what their individual needs were within a neurodiversity-affirmative framework. Crompton et al. describe interviews about the post-diagnostic phase for autistic adults, discussing peer support and community connection. Autistic adults reflected on the benefits of spending time with other autistic people, especially within the post-diagnostic period. The ease and mutual understanding experienced within an autistic-only space may provide more comfortable support for autistic people following diagnosis than support provided by non-autistic people, and help autistic people to build resilience to manage living in a majority non-autistic world.

Access to diagnosis and post-diagnostic support can be crucial in improving wellbeing for autistic people. Many autistic people experience misdiagnosis prior to being identified as autistic, which Iversen and Kildahl attribute to diagnostic overshadowing and a lack of autism specific expertise in mental health services. In their case report, they describe a patient who experienced misdiagnosis, which led to inappropriate psychopharmacological intervention. Once he was identified as autistic, treatment for his mental health difficulties were adapted and his quality of life improved, with him citing his autism diagnosis as a positive experience. The positive impact of an autism diagnosis was partially supported by findings from Corden et al. who conducted a mixed-methods exploration of the impact of diagnosis on identity. Time since diagnosis impacted on autistic personal identity, with people diagnosed more recently expressing more dissatisfaction with their identity compared to those for whom more time had passed. Qualitative data from this study suggested that the post-diagnostic adjustment period was emotionally fraught, and people found support throughout this period was often lacking.

Developing effective support for autistic people should be underpinned by understanding factors which impact on autistic quality of life across the lifespan. Phung et al. report findings from interviews with young people (aged 8–18) about the experience of burnout, inertia, meltdowns and shutdowns (BIMS). They identify the need for a more compassionate approach from trusted adults in supporting them during their experiences of these complex phenomena. These findings have important implications, given the prevalence of mental health difficulties reported by autistic adults later in life. Roestorf et al. found that over two thirds of autistic adults report physical and mental health difficulties in a longitudinal exploration of the relationship between mental health and quality of life outcomes. Two studies in this special issue focused on how application of knowledge about autistic mental health and support can improve outcomes for autistic university students. Cheriyan et al. found that autistic university students desired the opportunity to develop career-focused skills alongside mental health support. These findings were further supported by Lucas et al. who found that autistic university students reported feeling ill-prepared for the transition out of university into a career and desired support for this transition that focused on both emotional and career-related factors. Together, these four studies emphasize the need for approaches which identify factors which lead to negative outcomes for autistic people across the lifespan, and provide compassionate support informed by the preferences of autistic people.

Three papers in this special issue focus on how the development of robust and effective support for autistic people is fraught with problems. Two papers focus on effective support for autistic communication. Davis et al. examine the evidence in support of concerns that bilingualism may contribute to cognitive and language delays in autistic children. Their findings suggest autistic bilingual people should have equal access to language learning opportunities, supported by practitioners with up-to-date knowledge about neurodiversity. Similarly, Heyworth et al. discuss polarizing attitudes toward a form of Augmentative and Alternative Communication (AAC) term “facilitated communication” (FC). They argue that research into FC would benefit from a more up-to-date approach including autistic participatory involvement, and the absence of ableist assumptions about communicative competence. The final paper focuses on the presence of undisclosed conflicts of interest (COIs) in the Applied Behavior Analysis (ABA) literature (Bottema-Beutel and Crowley). ABA is frequently recommended as an intervention for autistic people (Xu et al., 2019), yet the evidence base for its efficacy is inconsistent (Sandbank et al., 2020) and Bottema-Beutel and Crowley found pervasive undisclosed COI's in the ABA literature, supporting the concerns of autistic people about the standard of ABA and associated interventions.

## Conclusions

The articles in this special issue highlight the evolving landscape of autism research, where increasingly work is starting to address the issues that autistic people and other stakeholders hold most valuable. Importantly, more than half of the articles include at least one autistic author, suggesting that calls for the involvement of autistic expertise in autism research are increasingly being answered. We hope that these advancements continue into the next decade and beyond.

## Author contributions

AP and CC wrote the initial draft of the manuscript. AS, CG, DP, FS, and SA-Y reviewed and edited the manuscript. All authors have agreed the final version.

## Conflict of interest

The authors declare that the research was conducted in the absence of any commercial or financial relationships that could be construed as a potential conflict of interest.

## Publisher's note

All claims expressed in this article are solely those of the authors and do not necessarily represent those of their affiliated organizations, or those of the publisher, the editors and the reviewers. Any product that may be evaluated in this article, or claim that may be made by its manufacturer, is not guaranteed or endorsed by the publisher.
